# A real-time object detection model for orchard pests based on improved YOLOv4 algorithm

**DOI:** 10.1038/s41598-022-17826-4

**Published:** 2022-08-08

**Authors:** Haitong Pang, Yitao Zhang, Weiming Cai, Bin Li, Ruiyin Song

**Affiliations:** 1 School of Information Science and Engineering, NingboTech University, Ningbo, 315100 China; 2 Intelligent Equipment Research Center, Beijing Academy of Agriculture and Forestry Sciences, Beijing, 100097 China

**Keywords:** Engineering, Imaging, Techniques and instrumentation

## Abstract

Accurate and efficient real-time detection of orchard pests was essential and could improve the economic benefits of the fruit industry. The orchard pest dataset, PestImgData, was built through a series of methods such as web crawler, specimen image collection and data augmentation. PestImgData was composed of two parts, PestImgData-1 and PestImgData-2. It contained 24,796 color images and covered 7 types of orchard pests. Based on the PestImgData and YOLOv4 algorithm, this paper conducted a preliminary study on the real-time object detection of orchard pests from 4 perspectives: transfer learning, activation function, anchor box, and batch normalization. In addition, this paper also visualized the feature learning ability of the detection models. On the basis of the above research, three improvement measures were adopted: the post-processing NMS algorithm was upgraded to DIoU-NMS, the training method was upgraded to 2-time finetuning training and the training data was enhanced. The performance of the improved model, F-D-YOLOv4-PEST, had been effectively improved. The mean average precision of F-D-YOLOv4-PEST was 92.86%, and the detection time of a single picture was 12.22 ms, which could meet the real-time detection requirements. In addition, in the case of high overlap area or high density, F-D-YOLOv4-PEST still maintained good performance. In the testing process of the laboratory and the greenhouse, including the wired network and the wireless network, F-D-YOLOv4-PEST could locate and classify pests as expected. This research could provide technical reference for the intelligent identification of agricultural pests based on deep learning.

## Introduction

In the process of fruit planting, orchard damage existed in multiple growth cycles of plant growth, and the quality and yield of fruits were therefore threatened. In order to minimize the economic losses, timely and accurate warning and identification of pests in orchards were needed^1,2^. Traditional orchard pest identification and classification mainly included the following 2 methods: the first method was through manual investigation; the second method was to collect and summarize the pests in the planting environment through light trapping or other means, and then conducted species identification through artificial methods. Both of the above 2 methods required manual intervention and had disadvantages such as poor objectivity, low efficiency, and poor real-time performance^3^. In addition, due to the diversity of pests and the similarity between species, manual identification required expert knowledge. An important research direction in artificial intelligence was computer vision based on deep learning^4–7^, which used image sensing devices such as cameras to capture visible objects in real life and performed intelligent calculation analysis. In fact, visual differentiation was also critical for pest identification. In recent years, scholars had also done a lot of research work in this field. Some researchers focused their work on pest classification, while others considered both classification and localization.

Many scholars^8–11^ realized the intelligent classification of pests based on the method of convolutional neural network, but there were still some problems: (1) The accuracy was generally not as expected and did not meet the conditions for practical use. (2) The amount of data was small. The generalization ability and robustness of the algorithm were not fully validated. The algorithm recognition ability in actual scenarios was unknown. (3) It did not have the ability to locate pests. (4) These algorithms did not meet real-time detection needs. In order to improve the classification accuracy of pests and solve the problem of localization, many scholars also carried out some valuable work. Some scholars^12–18^ introduced the classic network architecture (Faster R-CNN, RCNN, YOLOv3, SSD, MobileNet) in the field of object detection into the task of intelligent identification and localization of pests, and achieved remarkable results. The classification accuracy of pests had been greatly improved. More importantly, these research could also detect the location of pests. But in fact, these studies still did not solve the first two types of problems mentioned earlier. The dataset of the research was still small, and the performance in actual scenarios was unknown. In 2020, Wang and other researchers designed and built a new type of pest identification and detection model by deeply fusing the semantic information (temperature, humidity, latitude and longitude, etc.) of the pest image with the convolutional neural network model^19^. In this paper, the auxiliary information such as pest growth information was fused into the network. The actual performance was good, but it was difficult to expand due to the characteristics of pests.

In summary, the orchard pest object detection technology based on deep learning had the advantages of higher accuracy and better mobility, but there were still some problems: ① The scale of the image dataset was small and could not satisfy the needs of training and optimization of the DNN model; ② The detection speed was mostly slow, difficult to meet the needs of real-time detection, and the mAP still needed to be improved; ③ Most object detection model tests were limited to high-resolution laboratory environments and there was a lack of tests in actual planting scenarios. This paper focused on the above problems and a series of experiments had been carried out in the process of pest object detection.

## Materials and methods

### Dataset construction

Experts from Google and Carnegie Mellon University attributed the significant advantages of deep learning on computer vision tasks to the following three reasons: high-capacity models, rapidly growing computing power, and the availability of large-scale labeled data^20^.

**Figure 1:**
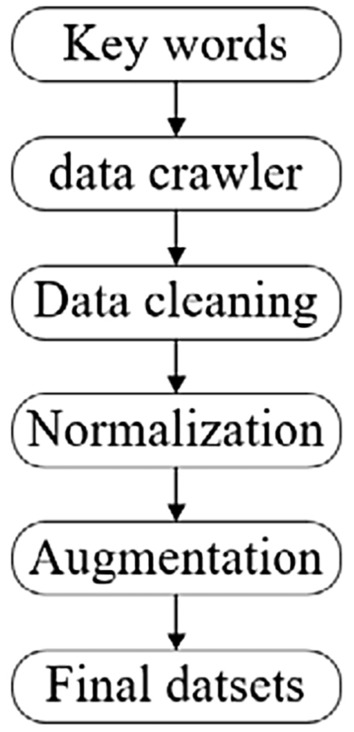
The acquisition process of PestImgData-1.

The training process of the CNN model was an iterative optimization process. The richer the image dataset, the more adequate the training of the model. However, if the amount of effective images was too small, it couldn’t be guaranteed that the model could obtain sufficient features during the training process. In terms of agricultural pests, it was very difficult to collect image datasets. The images used by most researchers came from laboratory image collection. The amount of images was small and could not meet the needs in this paper. Based on the above reasons, this paper used two methods to construct an RGB image dataset of orchard pests: PestImgData, which were web crawler (PestImgData-1) and laboratory specimen image collection (PestImgData-2). The process of building PestImgData-1 was shown in Fig. 1.

There were 7 types of target orchard pests, namely Cicadidae, Gryllotalpa spps, Scarabaeoidea and Locusta migratoria manilensis, Cerambycidae, Buprestidae and Hyphantria cunea. There were two searching keywords for each type of orchard pests: Chinese name and Latin scientific name. For each search keyword of each type, we searched its corresponding images on the three popular Internet search engines (Google, Bing and Baidu). Then, we crawled all kinds of images and merged them according to the category information. However, the preliminary image dataset of orchard pests obtained through the above methods still had the following problems: ① Many images were dirty data; ② The images obtained by multiple search engines and multiple keywords had a high degree of overlap and the redundant data needed to be eliminated; ③ The format, size and other characteristics of the images were inconsistent and needed to be normalized.

In order to solve the above problems, we used manual selection and normalization to filter all existing images. The size of the images was normalized to 320 × 320 × 3 and the format was unified to *.jpg*. The statistics of the effective image dataset were shown in Table 1 (before data augmentation).

**Table 1:** Statistics of PestImgData-1.

	Number of each category		Total	
	Before data augmentation	After data augmentation	Before data augmentation	After data augmentation
Cicadidae	433	3890	2500	22,481
Gryllotalpa spps	403	3620		
Scarabaeoidea	288	2592		
Locusta migratoria manilensis	354	3186		
Cerambycidae	343	3083		
Buprestidae	410	3690		
Hyphantria cunea	269	2420		

**Figure 2:**
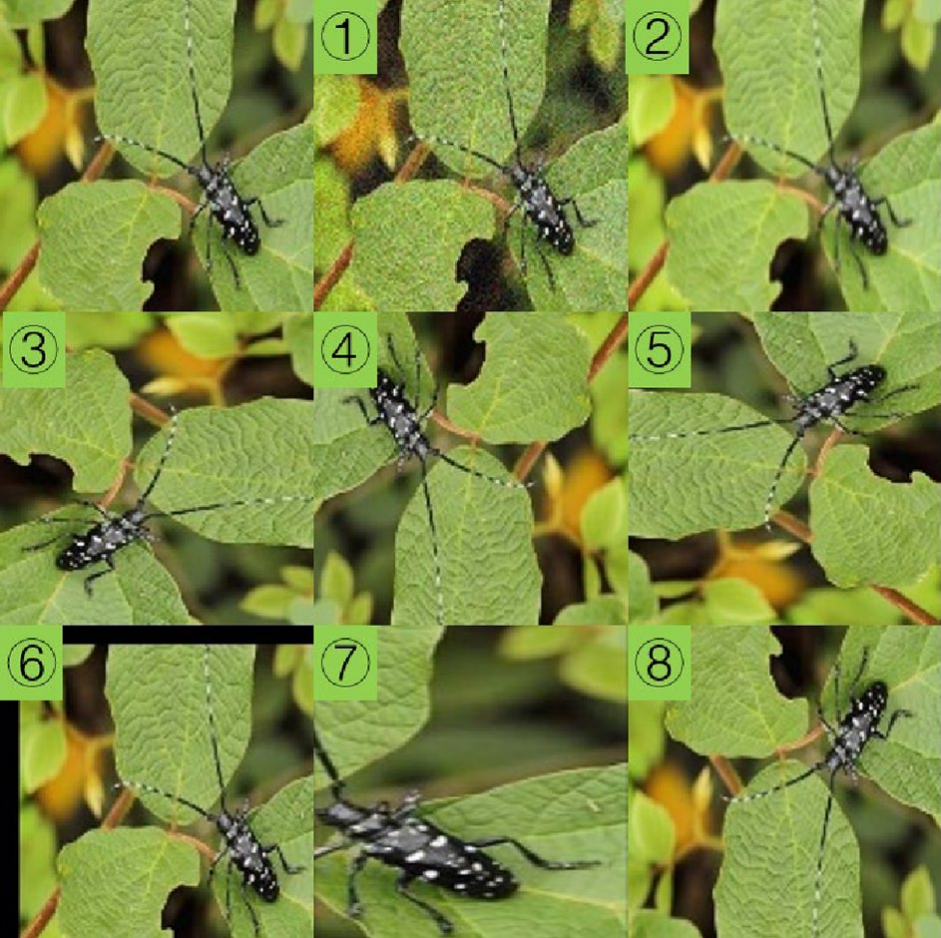
Data augmentation. *Note*: The first picture was the original. The serial number was consistent with the description in the above text.

In order to meet the needs of deep learning, we employed the data augmentation strategy. The purpose was to enlarge the existing image datasets through a series of technical means without generating data with essential differences. The main data augmentation methods adopted were as follows: ① noise; ② blur; ③ rotation (90); ④ rotation (180); ⑤ rotation (270); ⑥ translation and cropping; ⑦ zoom and stretch; ⑧ mirror inversion; ⑨ a combination of the above methods. A schematic diagram of various data augmentation methods was shown in Fig. 2. After data augmentation, the statistical information of the effective pest images was shown in Table 1 (after data augmentation).

The process of obtaining pest images through the method of specimen image collection was: ① chose the collection equipment and collection method; ② acquired preliminary image data; ③ random cropping and image normalization; ④ data augmentation. The number of pest specimens was as follows: Cicadidaes (6), Gryllotalpa spps (5), Scarabaeoidea (8) and Locusta migratoria manilensis (8), Cerambycidae (8) and Buprestidae (5). The image acquisition equipment was Jierui Microcom 1080P (approximately 1.84 million pixels) wide-angle 140-degree distortion-free industrial camera HF-869, which was connected to the computer via USB2.0. In the image acquisition process, additional objects in natural scenes such as branches and leaves were added to simulate the real environment. The image normalization method and data enhancement method were basically the same as those of PestImgData-1.

The statistical information of PestImgData-2 was shown in Table 2.

**Table 2:** Statistics information of PestImgData-2.

Orchard pests	Number	Total
Cicadidae	346	2267
Gryllotalpa spps	308	
Scarabaeoidea	342	
Locusta migratoria manilensis	315	
Cerambycidae	298	
Buprestidae	306	

**Figure 3:**
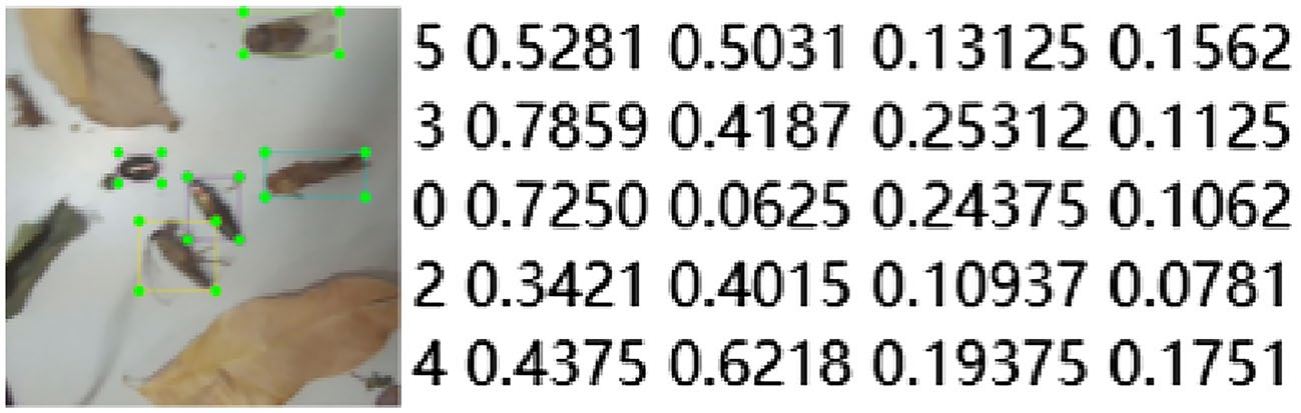
Schematic diagram of image annotation and coordinate information.

This research was based on supervised learning, so effective image annotation information was essential. In this study, an open source tool (LabelImg) was used to annotate the color images orchard pests. The annotation diagram was shown in Fig. 3.

In this section, the PestImgData-1 and PestImgData-2 were respectively annotated. So far, PestImgData had been successfully built. The statistical information of PestImgData was shown in Table 3.

**Table 3:** Statistics information of PestImgData.

Orchard pests	Number	Total
Cicadidae	4236	24,396
Gryllotalpa spps	3928	
Scarabaeoidea	2934	
Locusta migratoria manilensis	3501	
Cerambycidae	3381	
Buprestidae	3996	
Hyphantria cunea	2420	

### Experimental environment

All the research in this article were carried out under the same hardware and software environment. The specific parameters of the experimental environment were shown in Table 4.

**Table 4:** Experimental environment.

Resources	Details
CPU	AMD Ryzen 5 3600
GPU	NVIDIA RTX2070 Super
RAM	16 GB
Framework	Darknet
Language	C++, Python 3.6

In addition, during the initial training and testing of the model, the numbers of training dataset, validation dataset, and test dataset were 17,985, 900, and 3596. The ratio of the training dataset to the sum of other datasets was about 4: 1. The ratio of test dataset to validation dataset was about 4:1.

### Preliminary orchard pest object detection model based on YOLOv4 algorithm

YOLOv4^21^ was the fourth generation of YOLO series object detection algorithms, and its performance had been greatly improved. The improvement of YOLOv4 object detection algorithm mainly included the following 4 aspects: ① input: mosaics and other improvement measures were added; ② backbone: CSPDarket53 without FC layer; ③neck: FPN (feature pyramid networks) and PANet (path aggregation network); ④ head: IoU loss was replaced with CIoU loss. With the above improved measures, YOLOv4 algorithm achieved 43.5% mAP on MS coco dataset and achieved ∼65 FPS real-time speed on Tesla V100.

Generally speaking, the introduction of transfer learning would optimize the performance of the object detection models, but there were relatively few studies in the field of orchard pest object detection. The performance of transfer learning and its optimization capabilities still needed to be further studied. Driven by the above problems, this section quantified the impact of the introduction of transfer learning by the ablation experiment: training a model with transfer learning strategy and without transfer learning strategy.

First, on the basis of the YOLOv4 model, the first 137 basic weight parameters in YOLOv4.weights were extracted through weight separation. Then, in the two cases of not using the pre-training weight file and using the pre-training weight file, two detection models were trained respectively, namely NO-T-YOLOv4-PEST and T-YOLOv4-PEST.

In order to ensure the accuracy of the research results, during the training process, the parameter settings of the two types of models remained the same. The basic parameter setting was as follows: the size of the network input was 320 × 320 × 3, the batch was 64, the subdivisions was 16, the momentum parameter was 0.949, the maximum number of iterations was 14,000, the learning rate strategy was steps, the initial value was 0.001, the scales was 0.1, and the two step values of the learning rate change were 11,200 and 12,600 respectively, angle rotation was 0, both the saturation value and the exposure value was 1.5, the hue value was 0.5, the mosaic data enhancement was turned on, the multi-scale training mode was turned on; batch normalization was added by default, the NMS strategy was set to greedy NMS, etc.

**Figure 4:**
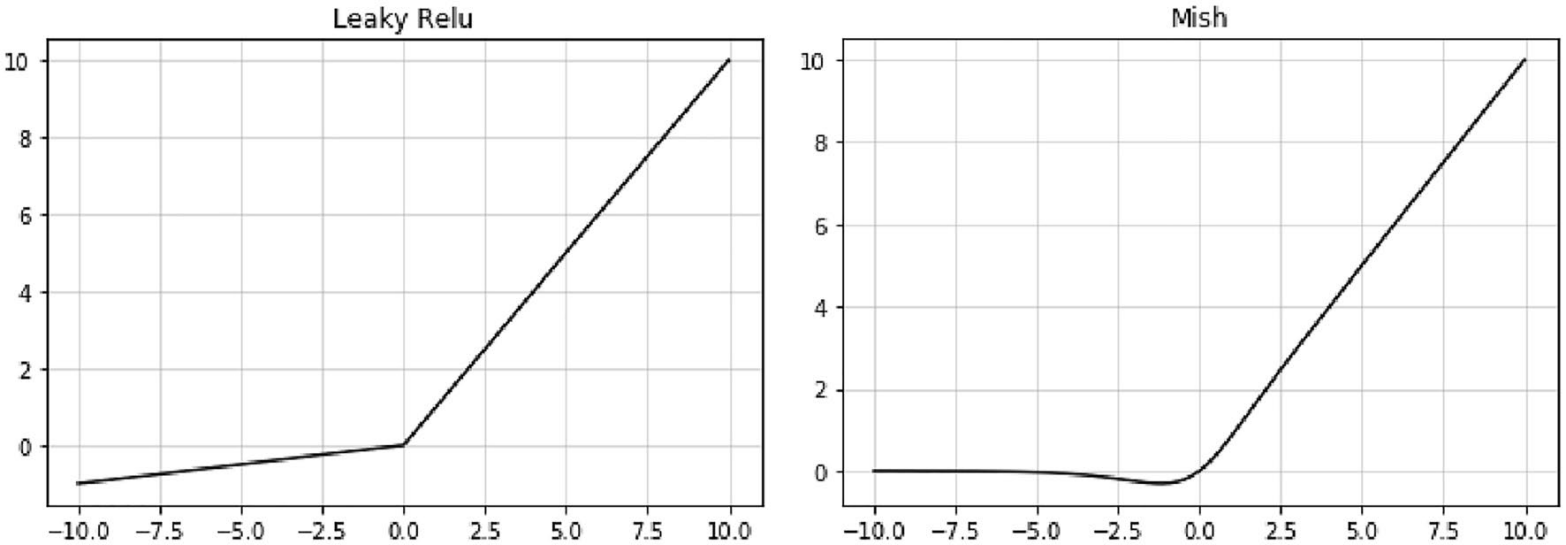
Schematic diagram of activation function.

The activation function was an important means to improve the non-linear feature acquisition ability of the network. Common activation functions included Sigmoid function, Relu function, Leaky Relu function and so on. In recent years, due to its excellent performance, the Leaky Relu function had brought different degrees of improvement to the model performance in many application scenarios. In addition, in 2019, some researchers proposed a new activation function: Mish function^22^, which had a smoother curve and better performance. The schematic diagram of Leaky Relu function and Mish function was shown in Fig. 4.

The mathematical expressions of Leaky Relu function and Mish function were as follows:

1 $${\text{Leaky}}\;{\text{Relu}}\;(x) = \left\{ {\begin{array}{*{20}l} {x,\quad x \ge 0} \\ {\lambda x,\quad x < 0 } \\ \end{array} } \right.$$

2 $${\text{Mish}}\left( {\text{x}} \right) = {\text{x}} \cdot {\text{tanh}}\left( {{\upzeta }\left( {\text{x}} \right)} \right)$$

Among them, λ is the slope control parameter of the Leaky Relu when $x$ is less than 0, which is generally a minimum value to avoid the problem of dead neuron. $\zeta \left( x \right)$ is the sum of the activation functions of softmax, and its mathematical definition is as follows:

3 $$\zeta \left( x \right) = ln\left( {1 + e^{x} } \right)$$

In the structure of YOLOv4, 133 activation functions were required, including Leaky Relu function, Mish function, and linear activation function. Mish activation functions were all located at the front end of the overall network structure (the feature extraction part). In order to study the influence of the Leaky Relu function and Mish function on the feature extraction part in the orchard pest detection model based on YOLOv4, two types of orchard pest detection models based on YOLOv4 were trained and the ablation experiment was set as follows: ① In the first 104 layers, set all the activation functions to the Leaky Relu function (ALL-LR-YOLOv4-PEST); ② In the first 104 layers, set all the activation functions to Mish functions (ALL-M-YOLOv4-PEST or T-YOLOv4-PEST model). In the model training process, the remaining basic parameter configuration was consistent with T-YOLOv4-PEST.

The essence of anchor box was to add the location information of possible objects to the model training process through some methods (manual formulation, algorithm clustering, etc.) before the training started. How to choose a highly representative anchor box in many rectangular boxes of different sizes was critical to the positioning accuracy and overall performance of the model. This paper used the K-means algorithm to generate anchor boxes.

The basic premise of using K-means algorithm clustering to generate anchor boxes was to have an image dataset containing annotation information (the center point coordinate values x and y of the real box, the width w and height h). The basic steps of the K-means algorithm were as follows: ① Gave the number of cluster center points K; ② Calculated the distance from each center point to the center point (x, y), as shown in formula (4), and the frame was classified to the closest center point d; ③ After all the real frames were classified, the center point was recalculated, as shown in formula (5); ④ Repeated the first two steps until the change in the center point was lower than the threshold.

4 $$d = 1 - IoU\left[ {\left( {x_{j} ,y_{j} ,w_{j} ,h_{j} } \right),\left( {x_{j} ,y_{j} ,W_{i} ,H_{i} } \right)} \right],\quad j \in \left\{ {1,2, \ldots ,N} \right\},i \in \left\{ {1,2, \ldots ,k} \right\}$$

5 $$W_{i}^{{\prime }} = \frac{1}{{N_{i} }}\sum w_{i} ,H_{i}^{{\prime }} = \frac{1}{{N_{i} }}\sum h_{i}$$

Among them, $x_{j}$ and $y_{j}$ are the center point coordinate information of the real frame, $w_{j}$ and $h_{j}$ are the width and height information of the real frame, $W_{i}$ and $H_{i}$ are the width and height of the anchor box, N is the number of real frames, and k is the number of cluster centers. In this section, based on two different types of anchor boxes, two models were constructed respectively. The two types of anchor boxes were: ① Universal anchor box (GEN-AB) based on COCO dataset; ② Custom anchor box (CUS-AB) based on PestImgData-1 orchard pest color image dataset. Their respective object detection models were GEN-AB-YOLOv4-PEST (T-YOLOv4-PEST) and CUS-AB-YOLOv4-PEST. The two types of anchor box data were as follows: GEN-AB (12, 16, 19, 36, 40, 28, 36, 75, 76, 55, 72, 146, 142, 110, 192, 243, 459, 401), CUS -AB (58, 73, 84, 177, 152, 105, 180, 199, 124, 304, 287, 149, 200, 333, 307, 227, 322, 323). In addition, in the process of training, the remaining basic parameter settings were consistent with T-YOLOv4-PEST.

Batch normalization (BN) was one of the important techniques in the network training process^23^. In the training process, due to the existence of forward propagation, the update of the shallow parameters would be continuously transmitted to the deeper layers, thereby changing the data input distribution, which was “Internal Covariate Shift”. BN corrected the data distribution of a single batch of data to solve the above problems. The specific steps were as follows: ① Calculated the mean value of the batch $\mu_{{\mathcal{B}}}$, as shown in formula (6); ② Calculated the variance of batch $\sigma_{{\mathcal{B}}}^{2}$, as shown in formula (7); ③ Normalized distribution adjustment, as shown in formula (8); ④ Translation and scaling, as shown in formula (9). Among them, $x_{i}$ is the ith input sample in the batch data, m is the total amount of batch data, $\hat{x}_{i}$ and $y_{i}$ are the data after normalization, $\epsilon$ is a very small positive number to prevent division by zero error, $\gamma$ and $\beta$ are scale transformation parameters.

6 $$\mu_{{\mathcal{B}}} = \frac{1}{m}\sum\limits_{i = 1}^{m} {x_{i} }$$

7 $$\sigma_{{\mathcal{B}}}^{2} = \frac{1}{m}\sum\limits_{i = 1}^{m} {\left( {x_{i} - \mu_{{\mathcal{B}}} } \right)^{2} }$$

8 $$\hat{x}_{i} = \frac{{x_{i} - \mu_{{\mathcal{B}}} }}{{\sqrt {\sigma_{{\mathcal{B}}}^{2} + \epsilon } }}$$

9 $$y_{i} = \gamma \hat{x}_{i} + \beta = BN_{\gamma ,\beta } \left( {x_{i} } \right)$$

This section explored the effect of BN on the performance of the orchard pest detection model based on YOLOv4 and the ablation experiment was set as follows: ① The network structure contained BN, which was named BN-YOLOv4-PEST (T-YOLOv4-PEST); ② The network structure did not contain BN, which was named NO-BN-YOLOv4-PEST. Since T-YOLOv4-PEST contained the BN layer, this section separately trained NO-BN-YOLOv4-PEST without the BN layer for comparative analysis. During the training process of the network model, the remaining network parameters were consistent with T-YOLOv4-PEST.

Computer vision technology based on deep learning had achieved remarkable results in many application scenarios, but the convolutional neural network model was still a “black box” model. In this paper, by extracting the feature maps of each level of the convolutional neural network model in the forward propagation process, the feature extraction ability of the network model was depicted by visual means. This section was based on the model T-YOLOv4-PEST to visualize its characteristic information in the network inference process. The process of feature visualization: ① Normalized the network inference feature map; ② Enlarged its value through the scaling factor and saved; ③ Mapped the file into a pixel matrix ④ Multi-channel pixel splicing. In the T-YOLOv4-PEST, the first four layers of output feature map information were visualized through the above ideas, and the size of the first four layers was: 320 × 320 × 32; 160 × 160 × 64; 160 × 160 × 64; 160 × 160 × 64.

### Pest object detection model based on the improved YOLOv4 algorithm

Non-maximum suppression (NMS) was one of the important methods in the object detection model to remove positioning frames with excessive overlap and inaccurate positioning accuracy. In the precious model training process, the NMS strategy in all models was Greedy-NMS and its algorithm steps were as follows: ① Input the positioning box $B = \left\{ {\left( {B_{n} ,s_{n} } \right)} \right\}_{{n = 1\;{\text{to}}\;N}}$, $s_{n}$ was the score of the corresponding positioning box, N was the number of positioning boxes, $D = \emptyset$; ② Selected the positioning frame M with the highest score in B; ③ Added M to D, and removed the relevant information of M from B; ④ Compared all the remaining positioning frames in B in sequence with their overlap with M. If it exceeded the set threshold, removed relevant information from B until the comparison of all positioning frames was completed; ⑤ Repeat the second, third, and fourth steps until B was an empty set.

Greedy-NMS had a major flaw: it only considered the overlap area of the two positioning frames. For the detection target with a high degree of overlap, there was a high probability that it would ignore some targets. In recent years, some researchers had proposed a new type of redundant positioning frame removal algorithm, the DIoU-NMS algorithm^24^, which had overcome the problem of single evaluation criteria in the Greedy-NMS algorithm. The Euclidean distance between the center points of the two positioning frames would be used at the same time, and its mathematical expression was:

10 $${\text{s}}_{{\text{i}}} = \left\{ {\begin{array}{*{20}l} {0,\quad DIoU\left( {{\text{M}},{\text{B}}_{{\text{i}}} } \right) \ge {\text{thresh}} } \\ {{\text{s}}_{{\text{i}}} ,\quad DIoU\left( {{\text{M}},{\text{B}}_{{\text{i}}} } \right) < {\text{thresh}} } \\ \end{array} } \right.$$

Among them, “thresh” is the set threshold, ${\text{s}}_{{\text{i}}}$ is the score of the ith positioning box, M is the current highest-scoring positioning box, ${\text{B}}_{{\text{i}}}$ is the ith positioning box, ${\text{DIoU}}\left( {{\text{M}},{\text{B}}_{{\text{i}}} } \right)$ is the DIoU distance of the two boxes. The specific calculation method of distance was shown in formula (11).

11 $${\text{DIoU}} = {\text{IoU}} - \left( {\frac{{{\text{d}}^{2} }}{{{\text{c}}^{2} }}} \right)^{{\upbeta }}$$

IoU is the intersection ratio of the two positioning frames, d is the Euclidean distance between the center points of the two boxes, c is the Euclidean distance of the distal ends of the two positioning frames, and β is the penalty parameter. It could be seen from formula (10) and formula (11) that when β was set to infinity, the DIoU-NMS algorithm would degenerate to the Greedy-NMS algorithm.

In the object detection technology based on deep learning, the amount of data played a decisive role in the final performance of the model. In this section, on the basis of PestImgData-1, the PestImgData-2 was added to the training dataset to enrich the features.

In the process of model training based on deep learning, there were generally two ways: training from scratch and using pre-trained model training. Through transfer learning, the convergence speed and detection accuracy of the orchard pest object detection model based on YOLOv4 had been improved. This research result showed that in the specific scenario of orchard pest object detection, transfer learning could bring significant performance gains. Therefore, in this section, based on the above experience, the model would be trained on the 2-time finetuning transfer learning to improve the performance of the orchard pest object detection model.

In this study, the final classification performance of the target orchard pests was one of the important performance evaluation indicators of the entire model. This paper used the PR (precision-recall) curve, AP (average precision) and mAP (mean average precision) to evaluate the classification performance of the model. Generally speaking, the predicted value of the model and true value might have the following possibilities, as shown in Table 5.

**Table 5:** Model prediction probability.

Reality\prediction	Positive	Negative
True	TP	FN
False	FP	TN

Precision represented the proportion of the correct part that was predicted to be true. Its mathematical expression was as follows:

12 $${\text{Precision }} = \frac{{{\text{TP}}}}{{{\text{TP}} + {\text{FP}}}}$$

Recall represented the proportion of the correct part that was truly true. Its mathematical expression was as follows:

13 $${\text{recall }} = \frac{{{\text{TP}}}}{{{\text{TP}} + {\text{FN}}}}$$

**Figure 5:**
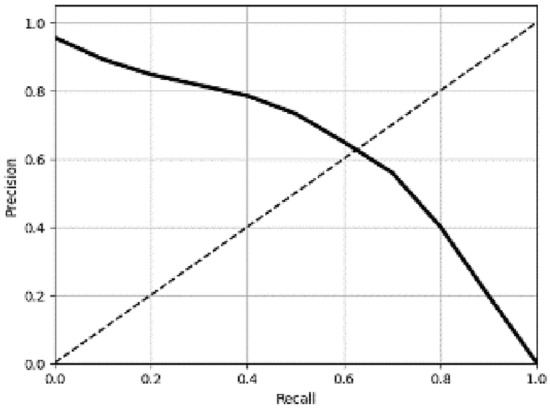
PR curve.

The abscissa of the PR curve was recall and the ordinate was precision. The basic schematic diagram was as follows:

As shown in Fig. 5, the closer the PR curve was to the point (1, 1), the better the performance of the model. Except for the PR curve, AP and mAP were also important indicators for model evaluation. Among them, AP represented the average precision of a certain type of detection object, and mAP was for multiple categories, which represented the average value of AP of multiple categories. In fact, the value of AP was the area enclosed by the PR curve and the two coordinate axes. The specific calculation formula was as follows:

14 $${\text{AP}} = \int\limits_{0}^{1} {{\text{p}}\left( {\text{r}} \right){\text{dr}}}$$

**Figure 6:**
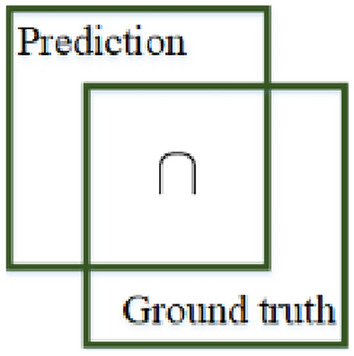
IoU.

For the object detection model, in addition to the classification accuracy of the detected target, the prediction accuracy of its location was also important. Intersection over union (IoU) was one of the important indicators to measure the positioning accuracy of the object detection model. As shown in Fig. 6, IoU measured the ratio of the intersection and union of ground truth and the prediction. The larger the value of IoU, the higher the positioning accuracy of the object detection model. Its mathematical expression was as follows:

15 $${\text{loU}} = \frac{{{\text{area}}\left( {{\text{Prediction}}} \right) \cap {\text{area}}\left( {{\text{Ground}}\;{\text{truth}}} \right)}}{{{\text{area}}\left( {{\text{Prediction}}} \right) \cup {\text{area}}\left( {{\text{Ground}}\;{\text{truth}}} \right)}}$$

In many scenarios, object detection tasks had higher requirements for detection speed, especially on dynamic detection tasks such as video detection. FPS (frames per second) was an important indicator to measure the detection speed of the object detection model. If the detection speed of the object detection model was 30FPS, it meant that it could detect 30 static images within 1 s, and the detection speed of a single image was about 1/30 ≈ 33 ms.

## Results and discussion

### Performance of preliminary orchard pest object detection models based on YOLOv4 algorithm

The performance of each orchard pest object detection model were shown in the Tables 6 and 7.

**Table 6:** The category detection AP of each model (IoU = 0.5).

Models	Orchard pests						
	Cicadidae	Gryllotalpa spps	Scarabaeoidea	Locusta migratoria manilensis	Cerambycidae	Buprestidae	Hyphantria cunea
T-YOLOv4-PEST	0.9085	0.9091	0.9038	0.904	0.9089	0.9025	0.9063
NO-T-YOLOv4-PEST	0.8986	0.9091	0.8791	0.8619	0.8983	0.9020	0.9012
ALL-LR-YOLOv4-PEST	0.9076	0.9091	0.9022	0.9036	0.9075	0.8988	0.9054
CUS-AB-YOLOv4-PEST	0.9081	0.9091	0.9031	0.9025	0.9083	0.9038	0.9043

**Table 7:** Basic performance indicators of each model.

Models	mAP@0.5	Detection time (ms)
NO-T-YOLOv4-PEST	0.8929	14.44
T-YOLOv4-PEST	0.9062	14.44
ALL-LR-YOLOv4-PEST	0.9049	12.22
CUS-AB-YOLOv4-PEST	0.9056	14.44

### Results and analysis of transfer learning research

**Figure 7:**
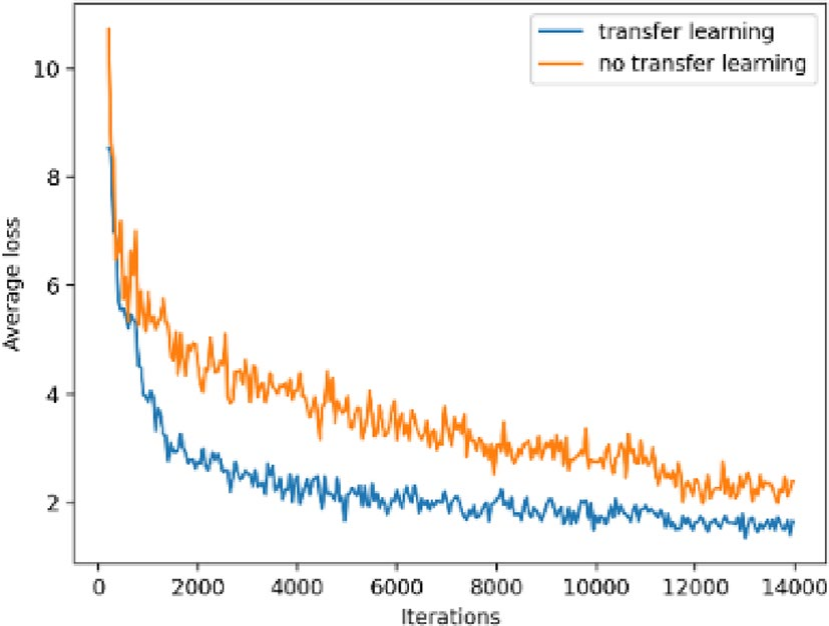
Average loss curves of training phase.

The loss function changes of NO-T-YOLOv4-PEST and T-YOLOv4-PEST during the training process were shown in Fig. 7.

Transfer learning speeded up the convergence of the model. After less than 2000 iterations, the average loss could drop below 3, while for the training process of non-transfer learning, it took about 6000 iterations to achieve the same degree of convergence. In addition, after 14,000 iterations, the loss value of T-YOLOv4-PEST could converge and stay below 2, which was significantly better than NO-T-YOLOv4-PEST.

**Figure 8:**
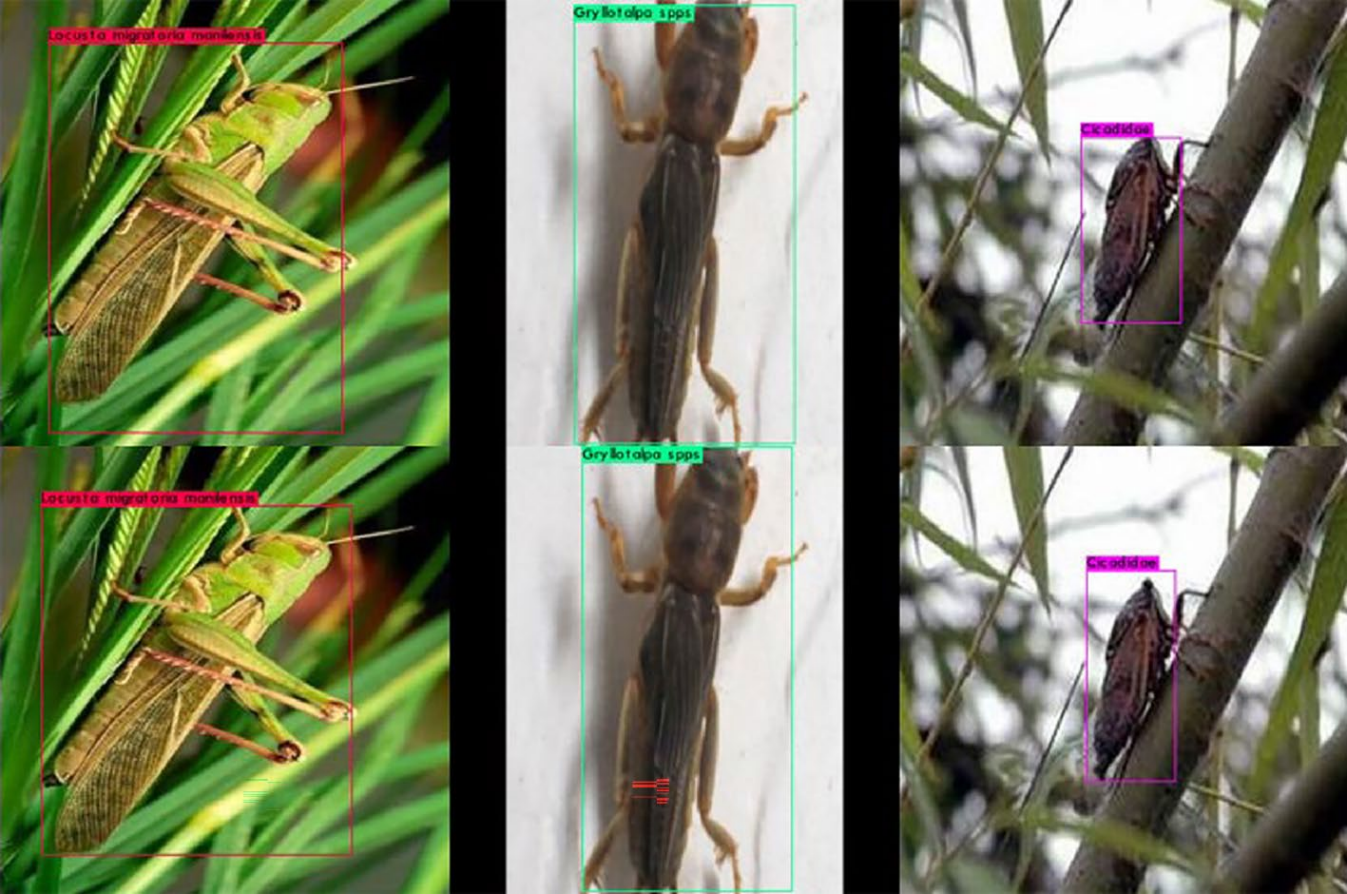
Test results (up: NO-T-YOLOv4-PEST; down: T-YOLOv4-PEST).

In summary, on the YOLOv4-based orchard pest object detection task and under the same parameter settings, transfer learning could improve the convergence speed of the model and reduce the lower limit of convergence. The test samples of the two models were shown in Fig. 8. It could be seen that although NO-T-YOLOv4-PEST and T-YOLOv4-PEST could also locate the pest position in the picture, the positioning frame of T-YOLOv4-PEST was better than that of NO-T-YOLOv4-PEST because the IOU area of the former was higher than that of the latter.

### Results and analysis of activation function research

From the experimental results in Tables 6 and 7, it could be concluded that the AP of the ALL-M-YOLOv4-PEST model was slightly higher than that of the ALL-LR-YOLOv4-PEST model in most cases. For mAP, the ALL-M-YOLOv4-PEST model was better than the ALL-LR-YOLOv4-PEST model by 0.1%.

**Figure 9:**
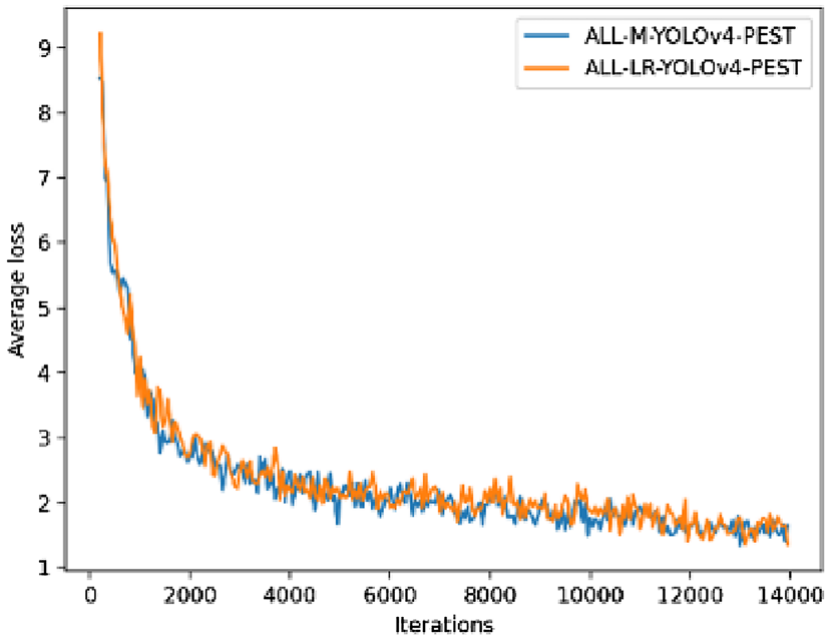
Average loss curves of training phase.

In addition, in the test process on 900 images, the ALL-LR-YOLOv4-PEST model took 11 s, while the ALL-M-YOLOv4-PEST took 13 s. The image detection time of the latter was 16.7% higher than the former. The changes in the average loss during the training of the two types of models were almost the same, as shown in Fig. 9.

Therefore, it could be concluded that on the PestImgData, the Mish activation function performed better than the Leaky Relu function, but the gap was very small. In addition, the detection time consumption of the former was higher than that of the latter.

### Results and analysis of anchor box research

**Figure 10:**
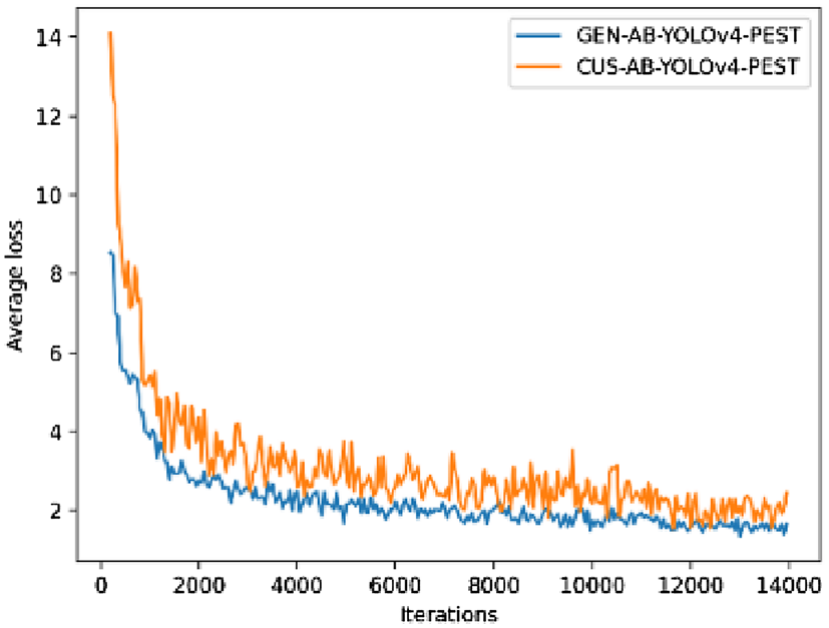
Average loss curves of training phase.

In the research process of anchor box, this paper constructed two models, NO-T-YOLOv4-PEST and T-YOLOv4-PEST. The loss curves of the two models were shown in Fig. 10.

In terms of category AP, it could be seen from Table 6 that GEN-AB-YOLOv4-PEST was better than CUS-AB-YOLOv4-PEST in the detection of Cicadidae, Scarabaeoidea, Locusta migratoria manilensis, Cerambycidae and Hyphantria cunea. The performance of GEN-AB-YOLOv4-PEST was equal to CUS-AB-YOLOv4-PEST in the detection of Gryllotalpa spps. The performance of GEN-AB-YOLOv4-PEST was weaker than CUS-AB-YOLOv4-PEST in the detection of Buprestidae. It could be concluded from Table 7 that in terms of overall performance, the mAP of GEN-AB-YOLOv4-PEST was higher than that of CUS-AB-YOLOv4-PEST, but the gap was just about 0.06%. The average loss of GEN-AB-YOLOv4-PEST was lower than CUS-AB-YOLOv4-PEST for most of the entire training process, and the final average loss was at a relatively low level. In addition, the fluctuation of the average loss value of CUS-AB-YOLOv4-PEST was significantly worse than that of GEN-AB-YOLOv4-PEST.

The reason why CUS-AB-YOLOv4-PEST had a worse performance than GEN-AB-YOLOv4-PEST could be mainly attributed to the following reasons: The anchor box in GEN-AB-YOLOv4-PEST was generated based on the COCO dataset clustering, while the anchor box in CUS-AB-YOLOv4-PEST was generated based on the PestImgData-1 clustering. Although the latter had more advantages in application scenarios than the former, the data volume of the COCO dataset was about 15 times that of PestImgData-1. The multiplied data volume was with better statistical significance, so the effectiveness of it was also higher.

### Results and analysis of BN research

**Figure 11:**
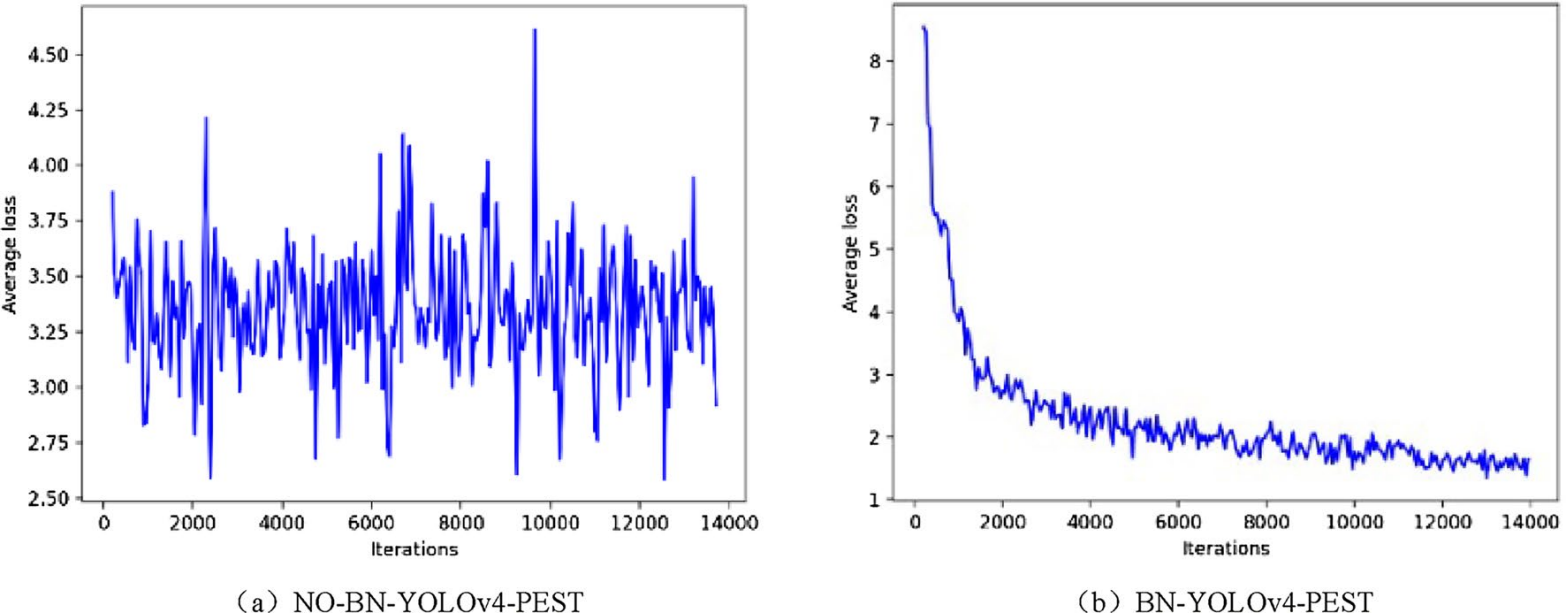
Average loss curves of training phase.

The average loss of NO-BN-YOLOv4-PEST and BN-YOLOv4-PEST during the training process were shown in Fig. 11.

During the training process, compared to the BN-YOLOv4-PEST model, the average loss of the NO-BN-YOLOv4-PEST model had been fluctuating, and there was no downward trend. The NO-BN-YOLOv4-PEST model did not effectively learn the basic features and depth features of the image data during the entire training process. In terms of the AP and mAP, the performance of BN-YOLOv4-PEST model was shown in the Tables 6 and 7. As for the NO-BN-YOLOv4-PEST model, because the feature information of the input images could not be effectively learned in the model training process, the AP and mAP was close to 0.

In summary, in the task of object detection in orchard pest based on YOLOv4, the existence of the BN layer was very important. The BN layer could enable the network model to overcome the negative impact of the input distribution deviation during the training process, thereby maintaining good network learning capabilities.

### Results and analysis of feature visualization research

**Figure 12:**
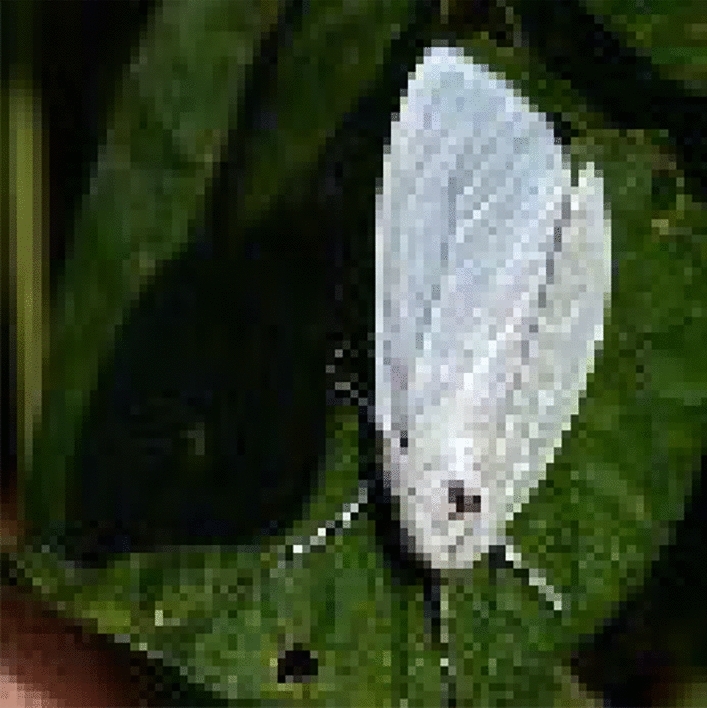
Original image (Hyphantria cunea).

**Figure 13:**
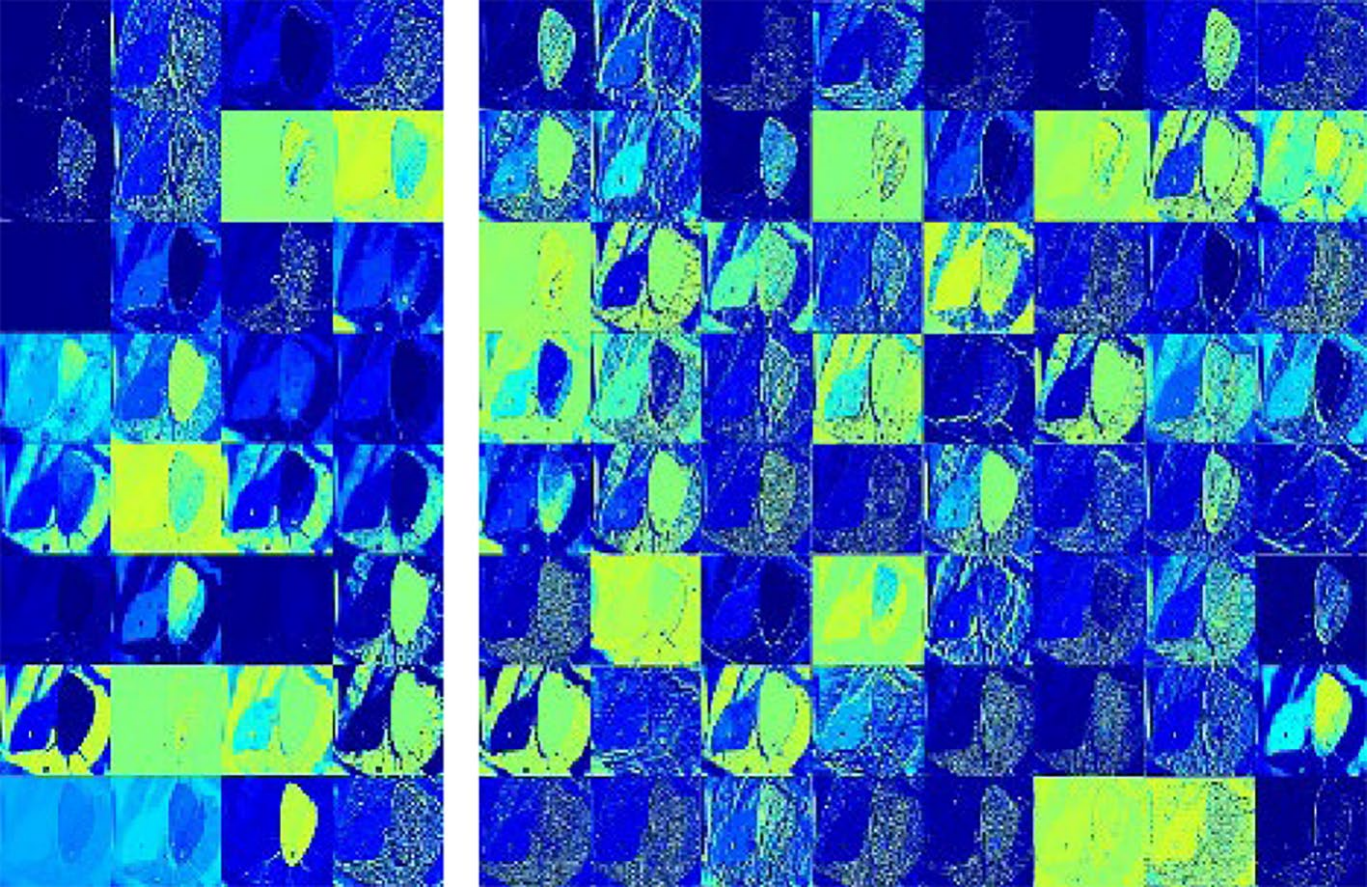
Feature visualization results (First layer and second layer). *Note*: The brighter the pixel, the higher the attention.

**Figure 14:**
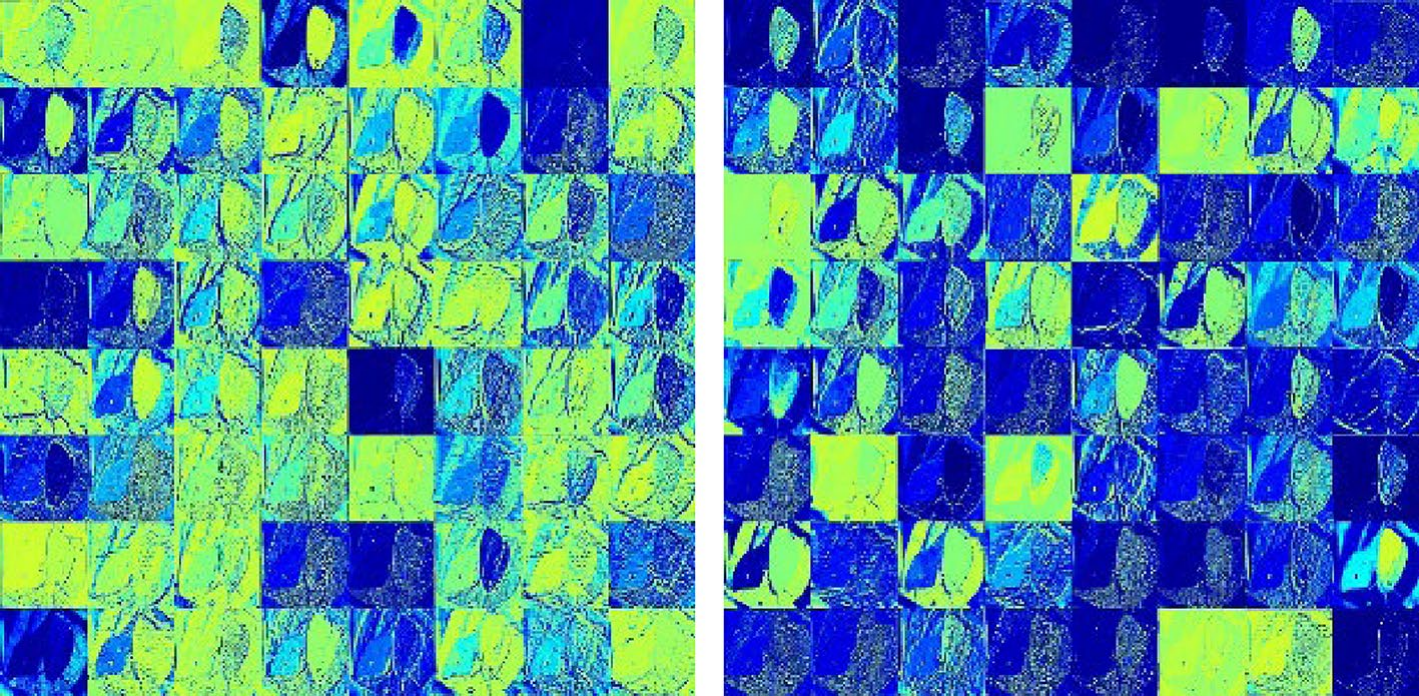
Feature visualization results (Third layer and fourth layer). *Note*: The brighter the pixel, the higher the attention.

In the research process of feature visualization, the image input of the model was shown in Fig. 12. The visualization results of the first four layers were shown in Figs. 13 and 14.

It could be seen from Figs.13 and 14 that with the gradual deepening of the network, the texture information, contour information and other content of the pest image continued to strengthen, and the convolutional neural network's ability to extract and summarize image features gradually increased. In addition, as the number of network layers deepened, the resolution of the image became smaller, and the feature information extracted by the convolutional neural network was also more representative. For example, the second, third, and fourth level feature maps were reduced to half the size of the input, and the overall outline and local information of the orchard pests were also richer.

### Results and analysis of basic model and improved model

By proposing three improvements to the basic model, a performance-upgraded version of the orchard pest object detection model was obtained. The performance comparison of the new and old models was shown in Tables 8 and 9.

**Table 8:** The category detection AP of each model (IoU = 0.5).

Models	Orchard pests						
	Cicadidae	Gryllotalpa spps	Scarabaeoidea	Locusta migratoria manilensis	Cerambycidae	Buprestidae	Hyphantria cunea
T-YOLOv4-PEST	0.9085	0.9091	0.9038	0.904	0.9089	0.9025	0.9063
NO-T-YOLOv4-PEST	0.8986	0.9091	0.8791	0.8619	0.8983	0.9020	0.9012
ALL-LR-YOLOv4-PEST	0.9076	0.9091	0.9022	0.9036	0.9075	0.8988	0.9054
CUS-AB-YOLOv4-PEST	0.9081	0.9091	0.9031	0.9025	0.9083	0.9038	0.9043
F-D-YOLOv4-PEST	0.9131	0.9992	0.9225	0.9168	0.9150	0.9144	0.9192

**Table 9:** Basic performance indicators of each model.

Models	mAP@0.5	Detection time (ms)
NO-T-YOLOv4-PEST	0.8929	14.44
T-YOLOv4-PEST	0.9062	14.44
ALL-LR-YOLOv4-PEST	0.9049	12.22
CUS-AB-YOLOv4-PEST	0.9056	14.44
F-D-YOLOv4-PEST	0.9286	12.22

**Figure 15:**
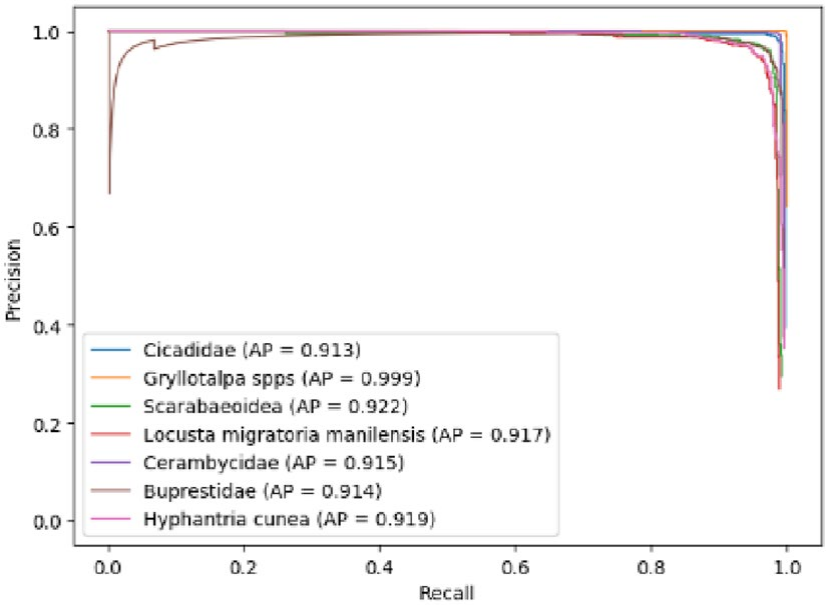
P-R curve of the F-D-YOLOv4-PEST.

It could be seen from Tables 8 and 9 that the performance of the F-D-YOLOv4-PEST was the best regardless of the single-category AP or mAP. The mAP of F-D-YOLOv4-PEST was 92.86%, which was much higher than other algorithm models. The addition of DIoU-NMS algorithm and finetuning training enhanced the learning and fitting ability of the network, and improved the overall performance of the model. In addition, the single-image detection speed of the model was 12.22 ms, which could meet the needs of real-time detection. The P-R curve of the F-D-YOLOv4-PEST on the test dataset was shown in Fig. 15.

**Figure 16:**
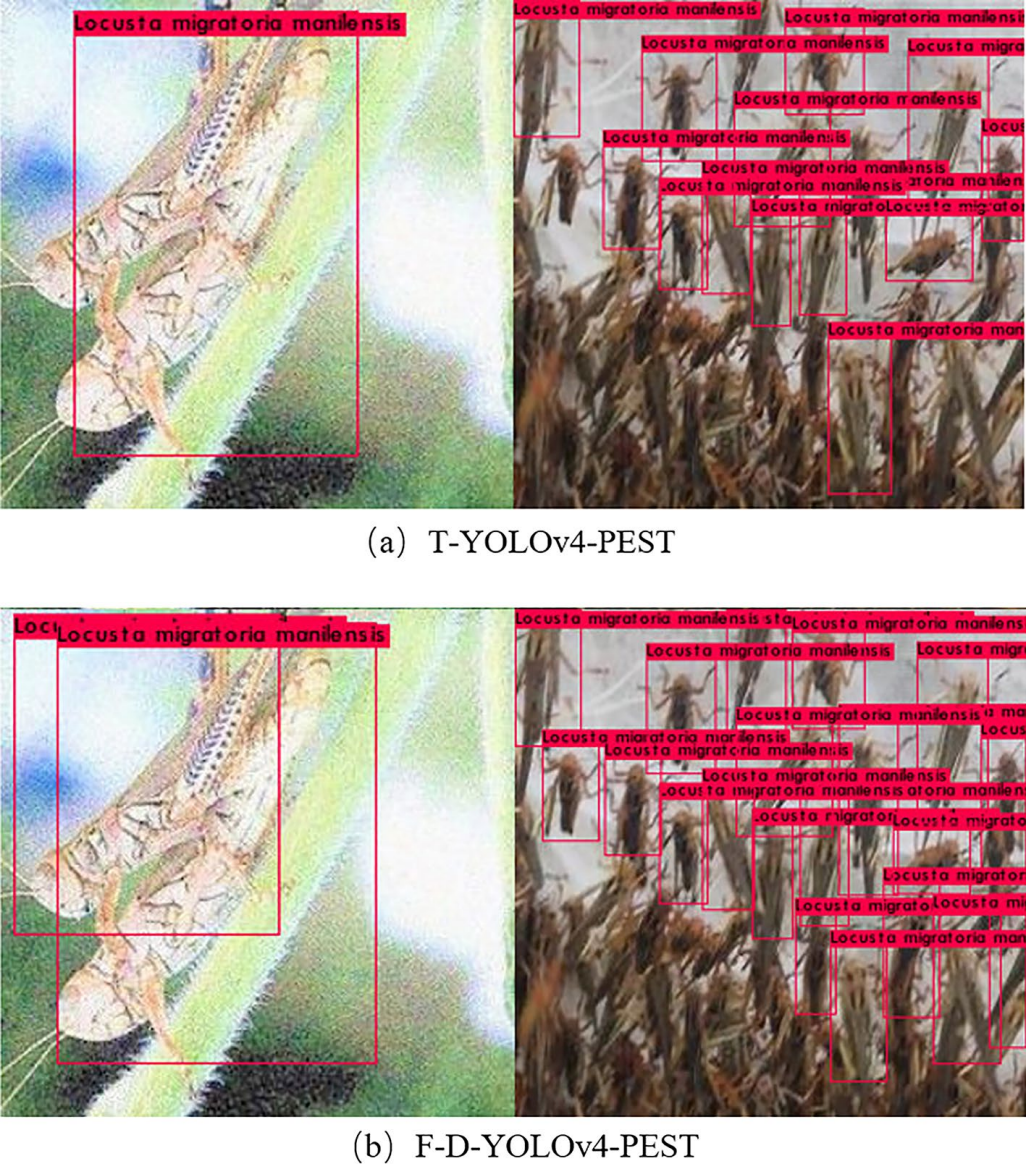
The actual detection results under high overlap and high density situations.

The actual detection results of the F-D-YOLOv4-PEST and the basic model T-YOLOv4-PEST on the test image were shown in Fig. 16. In the detection tasks of overlapping objects and high-density objects, the former had better performance than the latter.

### Results and analysis of the improved model and other classic object detection models

In order to comprehensively test the performance of F-T-YOLOv4-PEST, this section also trained 7 kinds of orchard pest object detection models based on Tiny-YOLOv4, YOLOv3, Tiny-YOLOv3, YOLOv2, Tiny-YOLOv2, VGG-Faster R-CNN and ResNet-Faster R-CNN (Tiny-YOLOv4-PEST, YOLOv3-PEST, Tiny-YOLOv3-PEST, YOLOv2-PEST, Tiny-YOLOv2-PEST, Faster R-CNN-PEST and Res-Faster R-CNN-PEST). In the training process of the above types of models, the settings of the basic network parameters remained the same as F-T-YOLOv4-PEST. The performance of various models was shown in Tables 10 and 11. It could be seen that the mAP of the F-T-YOLOv4-PEST was the best and the detection speed of F-T-YOLOv4-PEST could satisfy the needs of real-time detection.

**Table 10:** The category detection AP of each model (IoU = 0.5).

Models	Orchard pests						
	Cicadidae	Gryllotalpa spps	Scarabaeoidea	Locusta migratoria manilensis	Cerambycidae	Buprestidae	Hyphantria cunea
Tiny-YOLOv4-PEST	0.9000	0.9086	0.874	0.8359	0.9017	0.8967	0.8974
YOLOv3-PEST	0.9078	0.995	0.8855	0.899	0.9078	0.9006	0.9025
Tiny-YOLOv3-PEST	0.8856	0.9085	0.8526	0.8063	0.895	0.8875	0.8881
YOLOv2-PEST	0.9044	0.9083	0.8888	0.8226	0.9059	0.8971	0.8943
Tiny-YOLOv2-PEST	0.7058	0.9023	0.6599	0.5541	0.7952	0.7310	0.7355
Faster R-CNN-PEST	0.6807	0.8822	0.5095	0.5084	0.7503	0.6136	0.7009
Res-Faster R-CNN-PEST	0.8501	0.919	0.7509	0.7128	0.8727	0.7737	0.8145
F-D-YOLOv4-PEST	0.9131	0.9992	0.9225	0.9168	0.9150	0.9144	0.9192

**Table 11:** Basic performance indicators of each model.

Models	mAP@0.5	Detection time (ms)
Tiny-YOLOv4	0.8878	2.22
YOLOv3	0.9140	11.11
Tiny-YOLOv3	0.8748	3.33
YOLOv2	0.8888	6.66
Tiny-YOLOv2	0.7263	2.22
Faster R-CNN-PEST	0.6637	44
Res-Faster R-CNN-PEST	0.8134	117
F-D-YOLOv4-PEST	0.9286	12.22

### Results and analysis in multiple scenarios

**Figure 17:**
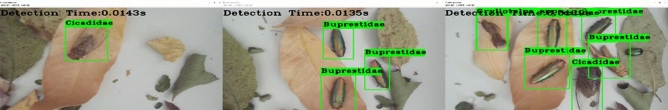
Test results in the laboratory with wired camera. *Note*: The first picture was a test result with a single insect, the second picture was a test result with insects and the third picture was a test result with multi-type insects.

The test in the laboratory scenario mainly included two parts: based on wired cameras and based on wireless camera. In the wired camera test process, the camera was connected to the computer via USB2.0; in the wireless camera test process, the IP camera data was read through the RTSP protocol. In the case of wired camera detection, the test results were shown in Fig. 17.

**Figure 18:**
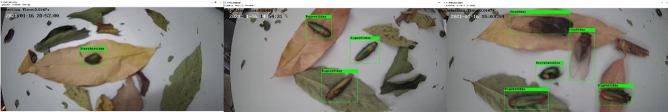
Test results n the laboratory with IP camera. *Note*: The first picture was a test result with a single insect, the second picture was a test result with insects and the third picture was a test result with multi-type insects.

From Fig. 17, it could be seen that F-T-YOLOv4-PEST performed better in real-time pest detection through wired cameras in laboratory scenarios and there was no time delay such as freezes and frame drops, and the detection time of the system could be s tably maintained within 15 ms. In the case of detection by IP cameras, the test results of F-T-YOLOv4-PEST were shown in Fig. 18.

The performance of real-time pest detection through IP cameras in the laboratory scenario was excellent. In addition, due to the introduction of the RTSP protocol and the delay of the network speed, the detection time consumption of the model had increased. But it could still be stably maintained within 16 ms to meet the requirements of real-time detection.

**Figure 19:**
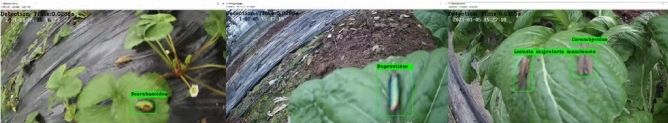
Test results in the greenhouse. *Note*: The first two pictures were a single-insect test result (*Scarabaeoidea* or *Buprestidae*) and the last picture was a multi-insect test result (*Locusta migratoria manilensis* and *Cerambycidae*).

The greenhouse involved in this study was located in Liangnong Town, Ningbo, Zhejiang Province, where various fruit crops such as strawberries and citrus were planted. The testing process under the greenhouse all adopted the IP cameras (under the Windows 10 environment). The test results of F-T-YOLOv4-PEST were shown in Fig. 19.

F-T-YOLOv4-PEST could classify and locate pest targets accurately, including single category and multiple categories. Since the system used RTSP video streaming for data acquisition, the detection speed was easily affected by network delays and fluctuates, but it still had real-time detection capabilities (within 16 ms). During the detection process, there was no obvious picture delay, frame rate attenuation, etc.

## Conclusions

This article had made some explorations on the intelligent identification of pests:

1. This paper build the PestImgData, which covered 7 types and 24,796 color images of orchard pests. All the images (RGB) in the dataset were already normalized and could be used for deep learning research.
2. Based on PestImgData, this paper conducted a series of studies based on YOLOv4, and finally generated an object detection model for orchard pests, namely F-D-YOLOv4-PEST. The model also had the ability to identify and locate pests, and could maintain a high accuracy of 92.86%.
3. The F-D-YOLOv4-PEST model proposed in this paper, with an accuracy of 92.86%, had good performance in the test indicators of different scenarios (laboratory and greenhouse) and had potential for practical application especially in scenarios with high real-time requirements.

For the intelligent detection of agricultural pests, the research in this paper had certain practical significance and reference value. However, further research will be needed for intelligent identification of pests in more complex scenarios.
